# Assessing Spatiotemporal and Quality Alterations in Paretic Upper Limb Movements after Stroke in Routine Care: Proposal and Validation of a Protocol Using IMUs versus MoCap

**DOI:** 10.3390/s23177427

**Published:** 2023-08-25

**Authors:** Baptiste Merlau, Camille Cormier, Alexia Alaux, Margot Morin, Emmeline Montané, David Amarantini, David Gasq

**Affiliations:** 1 ToNIC, Toulouse NeuroImaging Center, Université de Toulouse, Inserm, Université Paul Sabatier, 31062 Toulouse, France; 2 ISAE, Centre Aéronautique et Spatial, Université de Toulouse, 10 av. E. Belin, 31055 Toulouse, France; 3 Department of Functional Physiological Explorations, University Hospital of Toulouse, Hôpital de Rangueil, 31400 Toulouse, France; 4 Department of Neurorehabilitation, University Hospital of Toulouse, Hôpital de Rangueil, 31400 Toulouse, France

**Keywords:** inertial sensor, motion capture, kinematics, upper extremity, stroke rehabilitation

## Abstract

Accurate assessment of upper-limb movement alterations is a key component of post-stroke follow-up. Motion capture (MoCap) is the gold standard for assessment even in clinical conditions, but it requires a laboratory setting with a relatively complex implementation. Alternatively, inertial measurement units (IMUs) are the subject of growing interest, but their accuracy remains to be challenged. This study aims to assess the minimal detectable change (MDC) between spatiotemporal and quality variables obtained from these IMUs and MoCap, based on a specific protocol of IMU calibration and measurement and on data processing using the dead reckoning method. We also studied the influence of each data processing step on the level of between-system MDC. Fifteen post-stroke hemiparetic subjects performed reach or grasp tasks. The MDC for the movement time, index of curvature, smoothness (studied through the number of submovements), and trunk contribution was equal to 10.83%, 3.62%, 39.62%, and 25.11%, respectively. All calibration and data processing steps played a significant role in increasing the agreement. The between-system MDC values were found to be lower or comparable to the between-session MDC values obtained with MoCap, meaning that our results provide strong evidence that using IMUs with the proposed calibration and processing steps can successfully and accurately assess upper-limb movement alterations after stroke in clinical routine care conditions.

## 1. Introduction

Instrumental assessment of movement characteristics is a crucial component of post-stroke upper limb rehabilitation and longitudinal follow-up, and it is being recommended as a complement to clinical assessment [1,2,3].

Movement analysis consists of the characterisation of various tasks mimicking everyday life, such as drinking from a glass or reaching for a switch. It looks at different spatiotemporal and quality characteristics of the movement such as duration, hand trajectory, smoothness, and trunk contribution to hand displacement [4,5].

Marker-based motion capture (MoCap) offers advantages in terms of accuracy, spatial resolution, and capturing complex movement details [6]. However, it has major drawbacks for implementation in routine care because it requires a cumbersome and expensive setup with several cameras [7,8], as well as somewhat tedious calibration steps [6]. Labelling is no trivial matter either because of the difficulty of identifying markers and the presence of parasites [9]. Conversely, inertial measurement units (IMUs) provide a practical and versatile solution for assessing movement even in clinical conditions, particularly in terms of portability, real-time monitoring, and long-term assessment capabilities. Numerous studies have demonstrated that IMUs have thus become increasingly popular in assessing movement in a wide range of applications [10,11,12,13], and have proven that IMUs can be valuable tools for assessing movement alterations in clinical conditions [14,15]. However, their accuracy remains to be challenged. Indeed, IMUs can suffer from sensor drift, which is the gradual accumulation of errors in sensor measurements over time [16]. This can result in inaccurate readings, especially during long-duration measurements. Also, the accuracy of IMUs heavily depends on their correct calibration. To obtain reliable data, calibration procedures are necessary to compensate for sensor biases and to align measurements with body segments [17]. This can result in inaccurate readings, especially during long-duration measurements. Finally, IMUs require sophisticated algorithms and analysis techniques to extract meaningful information [18]. Interpreting the data accurately and extracting clinically relevant insights can be challenging, especially for complex movement patterns or in diverse clinical populations. Hence, validating the accuracy and reliability of IMU-based assessments across different clinical conditions and populations is an ongoing challenge. Establishing standardised protocols and benchmarks for the use of IMUs in specific clinical applications is essential for ensuring consistent and comparable results, especially in the context of routine care or home-based rehabilitation after stroke.

With regards to the above limitations to obtain informed outcomes using IMUs, it is important to consider that the most widespread approach when assessing upper limb kinematics is to compute the orientations of the segments of the limbs [19,20,21,22,23]. There are several drawbacks to this approach because it requires an extra step of sensor-to-segment calibration [24] that would increase fatigue for the subjects and jeopardise the feasibility on the one hand, and it is particularly sensitive to soft tissue artefacts on the other hand [25]. Furthermore, a kinematic model is needed to calculate the trajectory and finally derive the spatiotemporal and quality movement variables. Only a minority of the papers using this method analysed the trajectory [23], and none of them compared the spatiotemporal and quality variables with those from a gold-standard system (i.e., the MoCap system).

In contrast, the dead reckoning (DR) method directly provides the trajectory of an IMU. It still requires computing the orientation of the sensor in a fixed frame, but then a double integration is carried out to obtain the position. Consequently, this method does not require any sensor-to-segment calibration or a kinematic model. However, this approach is particularly sensitive to drift errors due to the integration steps, hence the need for offline calibration steps and other correction techniques [16]. Cahill-Rowley et al. used inertial sensors in combination with the DR method on healthy adults and toddlers and compared the spatiotemporal and quality variables with those from MoCap. Despite accurate results regarding several variables such as the peak velocity, the agreement on the index of curvature was poor and neither smoothness nor trunk compensation were investigated [26]. This remains, to our knowledge, the sole study comparing spatiotemporal and upper limb movement quality variables from IMU and MoCap systems.

In summary, IMUs are relevant motion analysis tools because of their ease of use, but they require sophisticated algorithms and analysis techniques to extract meaningful information. There are few data on their usability in routine care from which spatiotemporal and quality variables can be collected, including those that are based on the study of hand and trunk trajectory to assess upper limb movement alterations in post-stroke subjects.

The aim of this study was to investigate the agreement between the spatiotemporal and quality variables obtained from IMUs and a gold-standard MoCap for assessing movement alterations of the upper limb in post-stroke subjects in the context of routine care. As a secondary objective, we assessed the contribution of some IMU processing steps in increasing the agreement with the gold standard.

## 2. Materials and Methods

### 2.1. Subjects

Data were included from 15 post-stroke hemiparetic subjects (14 hemiparetic on the right and 1 on the left) assessed in routine care during their routine follow-up medical consultation or while they were hospitalised in rehabilitation. The detailed characteristics of the subjects are reported in Table 1. The subjects included 8 males and 7 females with a median age of 53 (range: 35–65) years, at a median of 3 (range: 1–36) months after stroke, and with a heterogeneous upper extremity function (median Fugl-Meyer Assessment motor component for the upper extremity: 62/66; range: 16–66).

### 2.2. Material

**Figure 1:**
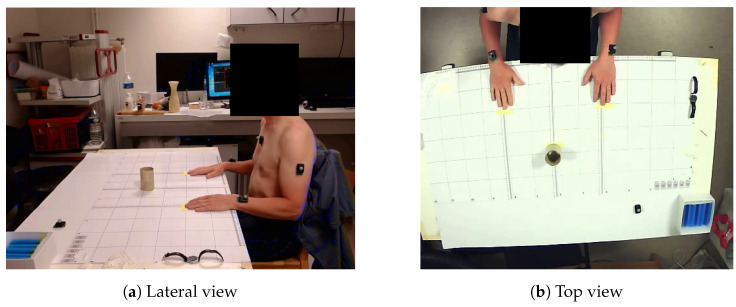
(**a**) Lateral and (**b**) top views of the experimental setup for the DRINK task (IMU sensors and reflective markers on the paretic arm and the non-paretic side were not used for this study). Subject is in the starting position.

The wearable IMU system consisted of Delsys Avanti Trigno sensors (Delsys Inc., Natick, MA, USA) placed on the subject, with one on the dorsal side of the paretic wrist, and the other one on the upper part of the sternum, as depicted in Figure 1. An IMU was composed with a 3-axis accelerometer (±16 g), a 3-axis gyroscope (±2000°/s), and a 3-axis magnetometer.

Motion capture, used as the gold standard, was carried out using an Optitrack system (model S250e, NaturalPoint, Corvallis, OR, USA) with eight cameras. One reflective marker was put on each inertial sensor.

The acquisitions were made with a sampling rate of 148 Hz for the IMUs and 250 Hz for the MoCap system.

### 2.3. Determination of Calibration Parameters

For each subject, before placing the IMU sensors, a static calibration [23] was performed by the rater. This procedure aimed to mitigate the influence of the variations in offsets and scale factors of the IMUs. The three IMUs were placed in a box designed so that they cannot move inside, which was then successively held on the horizontal table in the 6 orientations $\left\{+x,-x,+y,-y,+z,-z\right\}$ with a pause of 5 s between each position. The offsets $a_{offset}^{w}$ and scale factors $k^{w}$ of the accelerometer were computed as follows:

(1) $$k^{w}=\frac{2}{a_{+w}^{w}-a_{-w}^{w}}\cdot g,$$

(2) $$a_{offset}^{w}=\frac{a_{+w}^{w}-a_{-w}^{w}}{a_{+w}^{w}+a_{-w}^{w}}\cdot g$$

For $w=\left\{x,y,z\right\}$ with g=9.80665 m·s${}^{-2}$ and $a_{\pm w}^{w}$, the mean value of the accelerometer is on axis *w* when the ±w axis of the IMU is aligned with the gravitational acceleration. Offsets of the gyroscope were the mean of the gyroscope output during stability periods (independently of the orientation). Determining scale factors for the gyroscope requires dynamic calibration that generally involves a speed controlled turntable [23], and this step was not considered for this study.

### 2.4. Procedure

#### 2.4.1. Subject Setup

After the calibration, the IMUs equipped with a reflective marker were placed on the subject. For the starting position, subjects were sat on a chair with their back against the backrest, their hands flat on the table, and their elbows flexed at 90°. The target (i.e., a glass for the *DRINK* task or a switch for the *LIGHT* task) was placed such that the wrist had to cover 80% of the subjects’ arm length from the starting position to the object, without engagement of the trunk.

#### 2.4.2. Synchronisation

After starting the record, a supplemental IMU sensor equipped with a reflective marker was quickly moved in the vertical plane to create a high and narrow velocity peak. This procedure made it possible to synchronise the signals from the two recording systems if necessary.

#### 2.4.3. Task Realisation

Subjects were then asked to perform two series of fifteen repetitions of the task with the paretic upper extremity, with pauses of around 2 s between two repetitions. For the first series, subjects performed the task with a free trunk, while for the second one, their trunk was held against the back of the chair by the examiner.

For the DRINK task, from the starting position, subjects were asked to reach and grab the glass on the table, bring it to their lips, put it back to its initial placement, and come back to the starting position. For the LIGHT task, from the starting position, they were asked to reach and tilt the switch and come back to their initial position.

### 2.5. Data Processing

**Figure 2:**
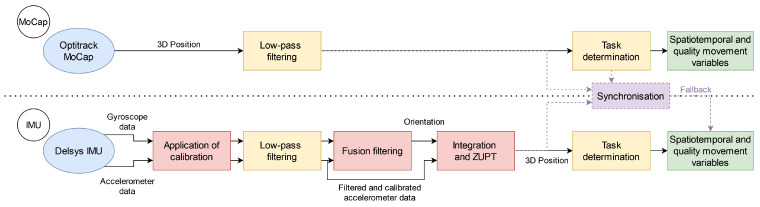
Processing steps for IMUs and MoCap data.

All the data processing was carried out under Matlab (MathWorks, Natick, MA, USA). Figure 2 shows the processing steps for the IMUs and MoCap data, and detailed explanations are provided in the next three sections.

### 2.6. MoCap Data Processing

Position data from each reflective marker were filtered using a low-pass fourth-order zero-phase Butterworth filter, with a cut-off frequency of 10 Hz.

### 2.7. IMU Data Processing

#### 2.7.1. Application of Calibration

Using the calibration parameters determined during the calibration procedure (Section 2.3), accelerometer and gyroscope data are calibrated with:

(3) $$a_{calib}^{w}=k^{w}\cdot a_{raw}^{w}-a_{offset}^{w}$$

(4) $$\omega_{calib}^{w}\;=\;\omega_{raw}^{w}\;-\;\omega_{offset}^{w}$$

where $w\;=\;\left\{x,y,z\right\}$.

#### 2.7.2. Filtering

Accelerometer and gyroscope data were then filtered using a low-pass fourth-order zero-phase Butterworth filter, with a cut-off frequency of 10 Hz.

#### 2.7.3. Fusion Filtering

Accelerometer data are collected in the IMU frame (noted as *d*) and need to be rotated in the constant laboratory frame (noted as *g*) whose z-axis is vertical and up.

Fusion filtering encompasses any method that obtains the orientation of the IMU from the accelerometer and gyroscope. Obtaining the orientation from the gyroscope is straightforward, as it provides the angular velocity so the orientation can be derived by integration. Consequently, errors increase during the measurement, meaning that this measure is only reliable for a short time span.

Conversely, the accelerometer is effective when computing the attitude for low-frequency movements as it allows us to determine the pitch and the roll from the relative orientation with the gravitational vector. The same principle can be applied to the magnetometer in combination with the magnetic field vector that provides information on the yaw, but because of magnetic disturbances in the laboratory room, the magnetometer was not used for this study [27].

The aim of the fusion filter is to fuse these two sources of information to provide the most accurate orientation. There are a lot of fusion filters presented in the literature with very distinct mathematical processes. Most of them, such as the complementary filter or the Madgwick filter [28], require some parameter tuning to work properly and may have an inconsistent performance depending on the characteristics of the movement.

For this study, we used the publicly available VQF filter [29] that did not require any parameter tuning. The output of the filter was given as a quaternion, which was then converted in the rotation matrix $R_{d}^{g}$.

#### 2.7.4. Integration and ZUPT

Given the rotation matrix $R_{d}^{g}$ and the (calibrated and filtered) outputs of the accelerometer $a_{d}$, we computed the acceleration in the global frame with:

(5) $$a_{g}=R_{d}^{g}a_{d}-g,$$

where $g={\left[0\;0\;9.80665\right]}^{T}$ m·s${}^{-2}$ is the gravitational acceleration.

This acceleration was integrated once using the trapezoidal rule to obtain the velocity. To mitigate the drift error, ZUPT (zero-update velocity) [16], based on the gyrometer norm, was implemented: stability periods were detected when the norm of the angular velocity from the gyrometer was under a certain threshold. The values of the thresholds were different for each IMU (0.07 rad/s for the wrist and 0.03 rad/s for the trunk) and were determined by trial and error. During those periods, the velocity was set back to 0.

Finally, the velocity was integrated a second time using the trapezoidal rule to obtain the final position vector.

#### 2.7.5. Influenceof the Processing Steps

To assess the influence of each processing step on the quality of the measurement, three scenarios (referring to the red steps in Figure 2) were considered:

1. Disabling the calibration (Section 2.7.1);
2. Using the Madgwick filter [28] (with parameter β=0.3 chosen by trial and error) as another fusion filter instead of the VQF filter (Section 2.7.3);
3. Disabling ZUPT (Section 2.7.4).

### 2.8. Approach Delimitation and Kinematic Variables

Approach phase delimitation was performed using the same algorithm for both the MoCap and IMU data, which is based on selecting the sagittal velocity peaks on the wrist IMU sensor/reflective marker:

- The examiner defined a positive velocity threshold for the peaks, which are then automatically selected.
- Irrelevant peaks were manually removed (i.e., duplicated peaks, failed attempts, etc.).
- The approach phase was selected (the performance of the task itself, bring the glass to lips and put it down or press the switch, has not yet been analysed).
- The start and end of the approach phase were defined by a tangential velocity threshold along the antero-posterior axis > and < at 1 cm·s${}^{-2}$. The start point was determined by parsing the velocity backward until it became lower than the threshold by at least 0.2 s (this duration threshold could be manually modified). Having an adaptable threshold was required due to working with subjects with severely altered movements who might stop in the middle of the approach. The end point was determined the same way by parsing the velocity forward.

**Figure 3:**
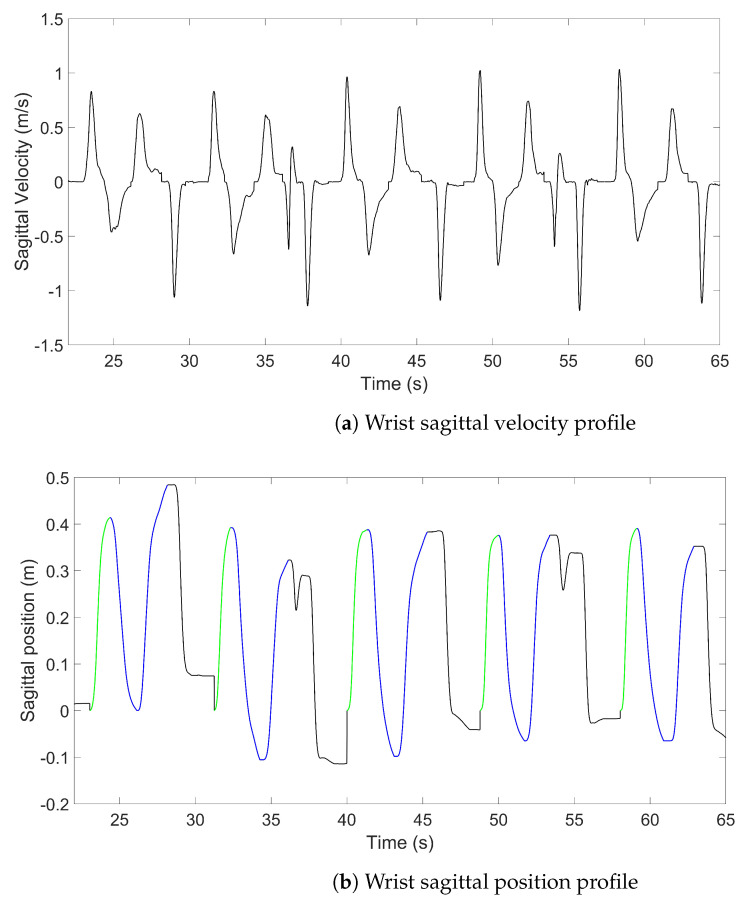
Sagittal velocity and position profiles for a sample sequence of 5 DRINK tasks. On the position profile, approach phases are green and the remainder of the task until the glass is put back at its initial position (not studied) is blue. Position is set back to zero at the beginning of each task.

An example of one series is shown in Figure 3. In case the signal of the IMU was altered in a way such that the phase delimitation is impossible, a fallback (outlined with a dotted line and purple in Figure 2) was envisaged by synchronising the delimitation of the IMUs and MoCap using the supplemental inertial sensor and selecting the phases from the MoCap data.

For each approach phase, four spatiotemporal and quality movement variables were computed:

- Movement time (MT) (in seconds): duration between the starting point and the end point of the approach.
- Index of curvature (IoC): ratio of the movement arc length to the shortest distance between the start and end points. Its value theoretically goes from 1 (perfectly straight trajectory) to +∞.
- Number of submovements (nSUB): number of sagittal velocity peaks; it is an integer from 1 to +∞, and higher values mean lower smoothness.
- Trunk contribution (TC) (in %): ratio of the sagittal length covered by the trunk to the sagittal length convered by the wrist.

### 2.9. Statistics

For each series and each subject, the first repetition was taken as a test run and was not considered. Moreover, as some recent papers suggest reducing the number of repetitions to between 3 and 5 [30,31], data from the 5 trials following the first one were averaged for the two systems. For the trunk contribution, we only included the free trunk series.

For each variable, we first checked the distribution of the absolute differences between the two systems and the value of the MoCap system using the Pearson correlation coefficient *r* and its *p*-value. A distribution was deemed heteroscedastic when the *p*-value was lower than 0.05, meaning that the differences tend to increase when the measured value increases [32]. Depending on the outcome of the test, we worked using the raw differences (when homoscedastic) or using the difference in % (when heteroscedastic). Following the Bland-Altman developments [32], we then computed the mean value ($\bar{d_{0}}$) and the standard deviation ($s_{0}$) and considered as outliers the series outside the $\bar{d_{0}}\pm T\times s_{0}$ interval (with *T* being the value obtained from the t-table with 29 (or 14 for the trunk contribution) degrees of freedom) [33]. With outliers excluded, we derived the ICC and its 95% confidence interval, as well as the mean difference (which corresponded to the bias) $\bar{d}$, its 95% confidence interval, and the minimal detectable change (which is identical to the limits of agreement following the Bland-Altman developments):

(6) $$\mathrm{mean}\;\mathrm{difference}\;95\%\;\mathrm{CI}:\bar{d}\pm T_{n-1}\times \frac{s}{\sqrt{n}}$$

(7) $$\mathrm{minimum}\;\mathrm{detectable}\;\mathrm{change}\;MDC=T_{n-1}\times s$$

where *n* is the number of series (with outliers excluded), *s* is the standard deviation, and $T_{n-1}$ is the value obtained from the t-table with n−1 degrees of freedom. A bias was considered systematic when its 95% confidence interval did not include zero.

## 3. Results

Among the 15 subjects, 13 of them were able to perform the DRINK task and 2 performed the LIGHT task (details in Table 1). All of the subjects were able to perform 15 repetitions of the selected task.

### 3.1. Agreement between IMUs and MoCap

Approach delimitation was successful for all of the subjects; some subjects, however, required the duration threshold of stability to be tweaked to include the entire movement: one subject made too short of pauses between repetitions, requiring lowering the threshold to 0.1 s, and two subjects had altered movement with pauses in the middle, so the threshold was increased to 0.3 s.

**Figure 4:**
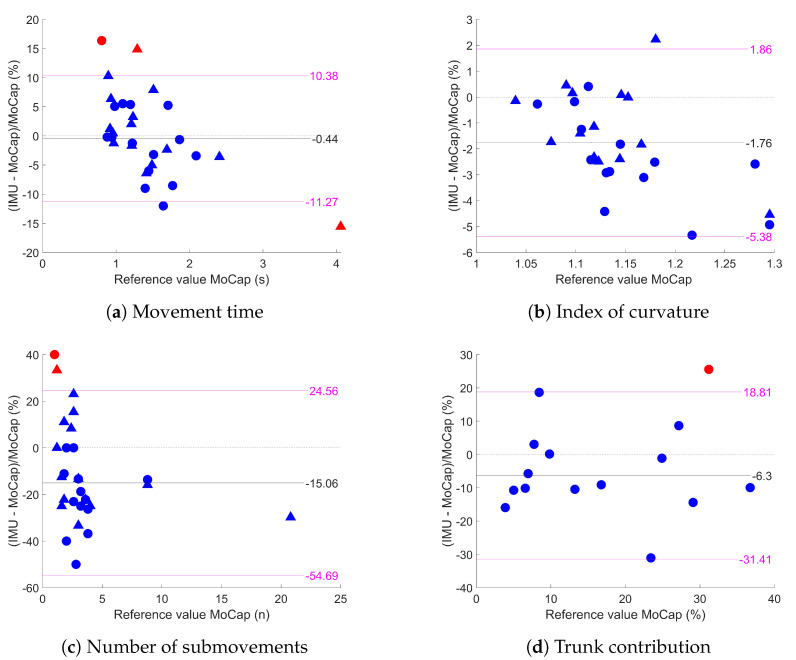
Bland-Altman plot between IMUs and MoCap for the four kinematic variables. Blue circles represent free trunk series and blue triangles represent maintained trunk series. Outliers are in red (except for the index of curvature where the two outliers were too different from the rest of the series and were cut from the plot). Black lines are the mean of the difference and pink lines are the limits of agreement.

Table 2 presents the mean values, ICCs, correlations, biases, and MDCs for the kinematic parameters, and Figure 4 shows the Bland-Altman plots for the four variables. For all of the variables, the distributions of the errors were heteroscedastic. For the index of curvature and the number of submovements, the systematic difference was significant (as zero was outside the confidence interval) but remained moderate for the number of submovements (−15.06%) and low for the IoC (−1.76%). The values of the MDCs varied among the variable: they were low for the MT (10.83%) and IoC (3.62%) but were higher for the TC (25.11%) and nSUB (39.62%).

### 3.2. Influence of the Processing Steps

The first observation was that whenever we removed any IMU processing step, the speed profile was too altered to reliably detect tasks, so the MoCap approach delimitation was used for this section. Velocity peaks were typically not detectable. Consequently, addressing the movement time for that section was irrelevant and we decided to use the same delimitation for the complete scenario to have the exact same processing in all scenarios.

**Figure 5:**
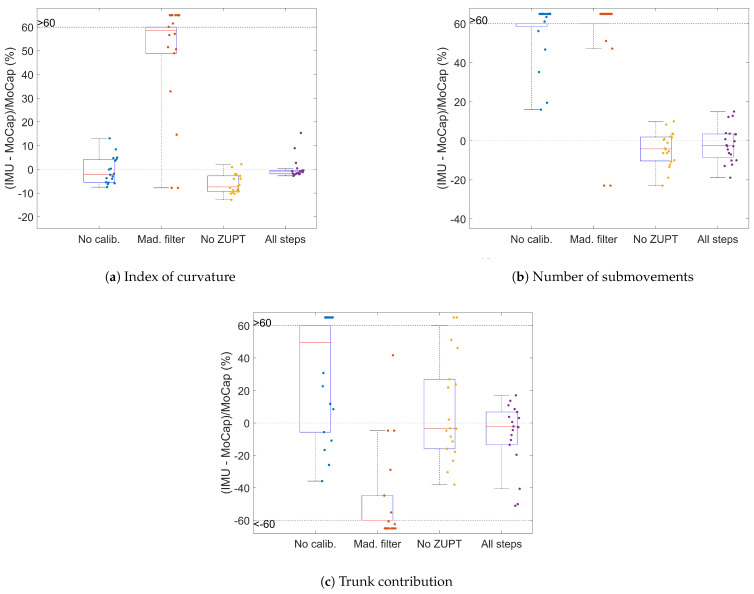
Boxplots of the errors between IMUs and MoCap with various steps of of processing; one point refers to one series.

Each of the three processing steps introduced a strong increase in the agreement. When disabling the calibration, significant systematic differences were introduced or increased for all of the kinematic variables, and MDCs became 1.3 to 4 times higher. The same observation was made when using the alternate fusion filter with a stronger degradation of the IoC. Finally, disabling ZUPT had a moderate influence on the IoC and the TC, and almost no influence on the number of submovements. Detailed statistics are provided in Table 3, and Figure 5 shows how the difference between the IMUs and MoCap evolves when altering the processing steps.

## 4. Discussion

In this work, we implemented a procedure and data processing to assess upper limb movement alterations in a clinical context with IMUs. We compared the spatiotemporal and quality variables obtained from the inertial system to the ones obtained from the MoCap gold-standard system. We also showed the importance and impact of signal processing steps on the quality of the measurement.

### 4.1. Agreement between IMUs and MoCap

Generally, the agreement between variables obtained from the IMU and MoCap systems was high, with variations among the variables. For the number of submovements, there was a significant negative bias (−15.06%), meaning that the inertial sensor reported fewer velocity peaks than MoCap, as well as had a high MDC (39.62%). The number of submovements is a very versatile variable as it may count very small peaks. For example, some of these peaks at the beginning or at the end of the movement could have been suppressed because the delimitation of the approach phase between the IMUs and MoCap was independent, or during very low-speed phases these peaks could have been considered as a period of stability by the ZUPT algorithm. Looking at the Bland–Altman plot, this negative bias (less than 3) mostly came from the lower values of the number of submovements with the IMU system, likely meaning that the inertial system missed 1 or 2 peaks at most. This limit of the number of submovements seems hard to overcome, and some recent papers suggest calculating other variables such as the SPARC to represent the smoothness of the movement [34].

There was a significant negative bias for the IoC with a small MDC, meaning that the inertial system tends to provide more direct trajectories. One plausible explanation is that small constant offsets still existed for the IMUs despite the calibration steps, adding some “straight” components to the trajectory and leading the IoC to slightly decrease. A major cause for changing offsets is temperature [35]. The calibration procedure was only performed once at room temperature and, due to the contact with the subject’s skin, the IMUs can become slightly hotter throughout the series. There is no simple mitigation for this as it is not possible to know the temperature of an IMU (unless the IMU itself contains a temperature sensor). Still, for the IoC, there were two strong outliers, as we could see in Figure 4, corresponding to the two series of the subject having the most altered movement. It was the only one with an IoC higher than 1.5 as well as with a very high number of submovements (>20 with the held trunk). For those series, the vertical component of the position was wrong and had an important positive offset plaguing the data. This explains why the IoC was underestimated for those two series (contrary to the three other variables that only depended on the sagittal component and which were quite accurate for those trials). If the most plausible explanation was a poorly executed calibration since the measured position was constantly higher than that of MoCap, the subject presented with highly altered movements with lower smoothness and longer approaches than the others, hinting that the performance of the IMUs might be lower with lower-performing subjects. This remains to be challenged as there were only two subjects in the study reporting a number of submovements higher than 5.

The trunk contribution had an MDC of around 25%. It was the only variable that depended on the two inertial sensors, and therefore was more sensitive to sources of errors. It was also much more sensitive to noise, as the trunk movements were most often slow and of a small amplitude.

It is relevant to interpret the level of measurement error computed between the IMU and MoCap systems with regard to the between-session test–retest measurement error obtained with a reference MoCap system for similar tasks [36,37,38], as shown in Table 4. For the MT, IoC, and TC, the between-system measurement errors were lower than those of the between-session ones. This result means that the measurement error is low enough with the use of IMUs to be used in clinical practice and replace the use of the MoCap system. This assumption has to be taken carefully, as there is little data about the reliability of spatiotemporal and movement quality variables, especially involving stroke subjects, with notable differences in terms of subject population, types of task, numbers of repetitions, and data processing (e.g., different ways to define approach phases) [36,37,38].

### 4.2. Influence of the Processing Steps

Each of the signal processing steps carried out with the IMUs showed its relevance in calculating a position that is sufficiently reliable to automatically delimit the approach phases of the tasks. Doing otherwise would involve manually selecting the task with a video or using another system (as we did for this part with MoCap) and would increase the time required in routine care. The choice of the VQF fusion filter instead of the Madgwick filter was the most crucial element associated with the greatest decrease in error margins (despite having tried different values for the Madgwick filter parameter β). By checking the outputs of the filters, we obtained errors up to 0.01 rad (i.e., 0.6°). This error seems very low, but the dead reckoning method we employed is particularly sensitive to orientation errors. For example, by considering a perfectly flat (without yaw or pitch), inactive, and calibrated IMU that would only measure the gravitational acceleration, then $a_{d}=\left[0\;0\;-1\right]{\;}^{T}g$. In this situation, if the fusion filter gives a yaw of 0.01 rad (instead of the actual 0 rad), then we can use Equation (5):

(8) $$a_{g}=R_{d}^{g}a_{d}-g=\left(\begin{array}{ccc} 1 & 0 & 0\\ 0 & \cos0.01 & -\sin0.01\\ 0 & \sin0.01 & \cos0.01 \end{array}\right)\cdot \left[\begin{array}{c} 0\\ 0\\ 1 \end{array}\right]-\left[\begin{array}{c} 0\\ 0\\ 1 \end{array}\right]\approx \left[\begin{array}{c} 0\\ -0.01\\ 0 \end{array}\right]g\approx \left[\begin{array}{c} 0\\ -0.1\\ 0 \end{array}\right]m/s^{2}$$

Such errors are unacceptable (leading to a 10 cm error in 1 s, and a 90 cm error in 3 s) for our application and explain why choosing an appropriate fusion filter is particularly important.

Calibration also played a major role in reducing the measurement error. However, the importance of the calibration may vary depending on the type of sensor given the recent advances and the development of IMUs. We chose to do the calibration before each subject to keep the same processing for each, but, especially in routine care, it may be worth lowering that requirement and investigating the impact of reducing the frequency of calibrations, even though carrying out the calibration only takes a few minutes.

The influence of ZUPT was moderate on the IoC and TC, and there was no significant modifications to the nSUB, which was expected as it was dependant on the acceleration (the number of velocity peaks being the number of times the acceleration crosses the zero line). There was still a small general decrease in the number of peaks, likely due to peaks being ignored during stability periods.

## 5. Conclusions

This study provides strong evidence for the relevance of using inertial sensors to assess spatiotemporal and movement quality alterations in the upper limb in post-stroke subjects in the context of routine care. By applying appropriate calibration to the sensors and data processing to mitigate the errors of the IMUs, we showed this method has high agreement with the MoCap system. We focused on using an easy and suitable procedure for implementation in routine care, meaning that replacing MoCap with wearable inertial sensors could lead to shorter subject care time and could be transposed in any environment outside laboratory rooms.

## Figures and Tables

**Table 1:** Subject characteristics.

Id	Age (Years)	Sex (F/M)	Time Since Stroke (Months)	Stroke Type (I/H)	Stroke Side (R/L), Location	Pre-Stroke Dominant Limb	Paretic Side (R/L)	FMA-UE (/66)	EmNSA-SS UE (/44)	Task Analysed
1	35	F	1	I	L, brainstem	R	R	63	44	DRINK
2	47	F	2	H	L, temporo-occipital lobar	R	R	66	44	DRINK
3	57	M	3	I	L, cortical MCA	R	R	40	40	DRINK
4	57	M	18	H	L, subcortical MCA	R	R	66	44	DRINK
5	61	M	36	I	L, lenticular	R	R	29	43	DRINK
6	47	F	2	I	L, capsulo-lenticular	R	R	16	43	LIGHT
7	65	M	1	I	L, cortical and subcortical MCA	R	R	66	44	DRINK
8	55	M	13	I	R, subcortical MCA	R	L	66	44	DRINK
9	53	M	8	I	R, pons and lower cerebellum	R	R	65	40	DRINK
10	58	M	8	I	L, cortical MCA	L	R	MD ${}^{*}$	MD ${}^{*}$${}^{*}$	DRINK
11	42	F	1	I	L, cortical and subcortical MCA	R	R	MD ${}^{*}$	44	DRINK
12	48	F	3	H	Median, pons	R	R	46	8	LIGHT
13	56	M	18	I	L, posterior cortical MCA	R	R	55	38	DRINK
14	52	F	1	I	L, subcortical MCA	R	R	32	44	DRINK
15	53	F	29	I	L, subcortical MCA	R	R	62	43	DRINK

Id: unique identification number, F/M: female/male, I/H: ischaemic/haemorrhagic stroke, R/L: right/left, FMA-M UE: Fugl-Meyer Assessment motor component for the upper extremity (/66), EmNSA-SS UE: Erasmus-modified Nottingham Sensory Assessment somatosensory component for the upper extremity, cortical/subcortical MCA: cortical/subcortical territories of middle cerebral artery, MD: missing data. ${}^{*}$ Fugl-Meyer score missing but Demeurisse motor index equal to 77/100 for both subjects. ${}^{*}$${}^{*}$ Hypoaesthesia (missing data related to phasic disorders).

**Table 2:** Agreement between IMUs and MoCap.

Variable (Unit)	r (*p*-Value)	Number of Outliers	MoCap Mean Value (SD)	IMU Mean Value (SD)	ICC [95%CI]	Bias [95%CI]	MDC
MT (s)	0.77 (<0.01 ${}^{*}$)	3	1.35 (0.40)	1.34 (0.36)	0.989 [0.977;0.995]	−0.44% [−2.61;1.72]	10.83%
IoC	0.92 (<0.01 ${}^{*}$)	2	1.14 (0.06)	1.12 (0.05)	0.935 [0.602;0.979]	−1.76% [−2.46;−1.06]	3.62%
nSUB (n)	0.91 (<0.01 ${}^{*}$)	2	3.71 (3.79)	3.01 (2.73)	0.955 [0.870;0.982]	−15.06% [−21.77;−8.36]	39.62%
TC (%)	0.69 (<0.01 ${}^{*}$)	1	15.69 (10.63)	14.52 (9.77)	0.983 [0.944;0.995]	−6.30% [−13.18;0.58]	25.11%

MT: movement time, IoC: index of curvature, nSUB: number of submovements, TC: trunk contribution. ${}^{*}$ Heteroscedastic distribution (p<0.05).

**Table 3:** Agreement between MoCap and IMUs under various processing scenarios.

Variable (Unit)	r (*p*-Value)	Number Outliers	MoCap Mean Value (SD) ^a^	IMU Mean Value (SD)	ICC [95% CI]	Bias [95% CI]	MDC ^b^
**No calibration**							
IoC	0.97 (<0.01 ${}^{*}$)	2	1.14 (0.07)	1.11 (0.06)	0.537 [0.050;0.780]	−2.85% [−5.10;−0.61]	11.69%
nSUB (n)	0.88 (<0.01 ${}^{*}$)	1	4.10 (5.72)	5.16 (3.53)	0.906 [0.791;0.957]	62.53% [41.76;83.31]	71.49%
TC (%)	0.92 (<0.01 ${}^{*}$)	0	16.74 (10.99)	18.26 (20.28)	0.706 [0.097;0.902]	8.47% [−25.23;42.17]	112.28%
**Madgwick fusion filter**							
IoC	0.95 (<0.01 ${}^{*}$)	2	1.17 (0.13)	1.80 (0.40)	−0.024 [−0.233;0.250]	55.23% [41.21;69.25]	48.53%
nSUB (n)	0.27 (0.15)	1	4.06 (5.64)	6.55 (6.14)	0.929 [0.457;0.979]	2.18 [1.41;2.95]	3.99 (98.28%)
TC (%)	0.36 (0.19)	0	16.10 (9.97)	6.31 (7.44)	0.557 [−0.258;0.858]	−9.79% [−14.07;−5.52]	15.13% (93.98%)
**No ZUPT**							
IoC	0.08 (0.67)	1	1.13 (0.05)	1.06 (0.05)	0.377 [−0.227;0.716]	−0.07 [−0.09;−0.05]	0.10 (8.85%)
nSUB (n)	0.74 (<0.01${}^{*}$)	1	3.16 (1.61)	2.62 (1.28)	0.941 [0.449;0.983]	−15.51% [−20.15;−10.86]	27.64%
TC (%)	0.19 (0.50)	1	18.71 (10.64)	17.24 (9.42)	0.981 [0.943;0.994]	−0.97% [−2.54;0.61]	5.35% (28.59%)

MT: movement time, IoC: index of curvature, nSUB: number of submovements, TC: trunk contribution. ${}^{*}$ Heteroscedastic distribution (p<0.05). ^a^ In the computation procedure, IMU and MoCap data are matched by subject and outliers are removed before computing the agreement. As a consequence, the MoCAP values in Table 3 are different among the processing steps and from those in Table 2 because the number of outliers are not the same under the various processing scenarios. ^b^ In the case of an absence of heteroscedasticity, the value of MDC expressed in % (MDC in original unit divided by the MoCap mean value) is given in brackets to allow the variables to be compared with each other.

**Table 4:** Comparison between the level of measurement error (MDC) computed for the IMU and MoCap systems and the between-session test–retest MDC obtained for a reference MoCap system.

	Task	MT (s)	IoC	nSUB (n)	TC (%)
IMU vs. MoCap	DRINK or LIGHT	10.83% (0.13 s) ^a^	3.62% (0.04) ^a^	39.62% (1.2) ^a^	25.11% (3.64%) ^a^
Patterson et al. [37]	Reach to grasp can (4 times)	0.42 s	0.08	–	– ^b^
Wagner et al. [36]	Point various targets (2 times each)	38.9–70.0%	7.4–28.9%	24.4–67.6%	–
Engdahl et al. [38]	Reach to grasp can (10 times)	–	0.15	0.50	–

^a^ Average MDC computed in original unit by multiplying the value of the MDC in percent by the mean IMU value. ^b^ Only a trunk displacement with an MDC of 41.1 mm was reported in the paper. Considering a covered wrist length of 35 cm, given the 80% arm length, this would lead to an absolute MDC in an original unit of about 12.0%.

## Data Availability

Data are available from the corresponding author upon reasonable request.
